# First Identification of HEV Subtype 3i in Human Hepatitis E Cases in Central Italy

**DOI:** 10.3390/v18070709

**Published:** 2026-06-27

**Authors:** Roberto Bruni, Michele Equestre, Valentina Curini, Barbara Camilloni, Silvia Bozza, Alessandro Graziani, Giovanna Picchi, Cinzia Marcantonio, Ludovica Arcopinto, Giulia Costanzi, Elida Mataj, Elisabetta Suffredini, Teresa Vicenza, Monica Borghi, Silvana Farneti, Orietta Staltari, Anna Rughetti, Elisabetta Madonna, Giuseppe Aprea, Cesare Cammà, Anna Rosa Garbuglia, Anna Rita Ciccaglione

**Affiliations:** 1 Unit of Viral Hepatitis and Oncovirus and Retrovirus Diseases, Department of Infectious Diseases, Istituto Superiore di Sanità, 00161 Rome, Italy; cinzia.marcantonio@iss.it (C.M.); ludovica.arcopinto@guest.iss.it (L.A.); giulia.costanzi@guest.iss.it (G.C.); elisabetta.madonna@iss.it (E.M.); annarita.ciccaglione@iss.it (A.R.C.); 2 Department of Neurosciences, Istituto Superiore di Sanità, 00161 Rome, Italy; michele.equestre@iss.it; 3 National Reference Center for Whole Genome Sequencing of Microbial Pathogens, Istituto Zooprofilattico Sperimentale Abruzzo-Molise “G. Caporale”, 64100 Teramo, Italy; v.curini@izs.it (V.C.); c.camma@izs.it (C.C.); 4 Microbiology and Clinical Microbiology Section, Department of Medicine and Surgery, University of Perugia, 06132 Perugia, Italy; barbara.camilloni@unipg.it (B.C.); silvia.bozza@unipg.it (S.B.); alessandro.graziani@collaboratori.unipg.it (A.G.); 5 Microbiology Unit, Santa Maria della Misericordia Hospital, 06132 Perugia, Italy; 6 Department of Life, Health and Environmental Sciences, University of L’Aquila, 67100 L’Aquila, Italy; giovanna.picchi.inf@gmail.com; 7 Department of Infectious Diseases, “Santa Rosa” Hospital, 01100 Viterbo, Italy; 8 Instituti i Shendetit Publik (ISHP), Rr. Aleksander Moisiu 80, 1001 Tirane, Albania; elidamata@yahoo.com; 9 Unit of Microbiological Safety and Foodborne Diseases, Department of Food Safety, Nutrition and Veterinary Public Health, Istituto Superiore di Sanità, 00161 Rome, Italy; elisabetta.suffredini@iss.it (E.S.); teresa.vicenza@iss.it (T.V.); 10 Istituto Zooprofilattico Sperimentale dell’Umbria e delle Marche, 06126 Perugia, Italy; m.borghi@izsum.it (M.B.); s.farneti@izsum.it (S.F.); 11 Immunotransfusion Medicine Unit, “San Salvatore” Hospital, 67100 L’Aquila, Italy; orietta.staltari@tin.it (O.S.); a.rughetti@tiscali.it (A.R.); 12 Food Hygiene and Technology Unit, Istituto Zooprofilattico Sperimentale Abruzzo-Molise “G. Caporale”, 64100 Teramo, Italy; g.aprea@izs.it; 13 National Institute for Infectious Diseases “L. Spallanzani” IRCCS, 00149 Rome, Italy; annarosa.garbuglia@inmi.it

**Keywords:** hepatitis E virus, HEV, subtype, phylogenetic analysis, p-distance, Sanger sequencing, WGS, NGS

## Abstract

HEV genotype 3 is classified into several subtypes. In Italy, 3f, 3c, 3e and, rarely, 3a subtypes are usually detected in human hepatitis E cases: the present study documents, for the first time, the detection of the 3i subtype, previously described in Italy exclusively from wild boars. Routine surveillance by short Sanger sequences highlighted five hepatitis E cases with unusual HEV subtype between 2019 and 2023 in a small geographical area in Umbria, Central Italy. Further characterization of the whole HEV genome by NGS was successful in three of them. Through phylogenetic and p-distance analysis, all the sequences could be classified as subtype 3i, and three of them proved to be highly related to some strains observed in wild boars sampled in the same geographical area. The 3i subtype had never been detected previously in humans in Italy; it is likely that increased local circulation of 3i-like strains in wild boars may have increased the chance for their transmission to humans. Investigation of the possible risk factors for HEV infection highlighted consumption of raw/undercooked products from wild boars/pigs in three cases. However, in the remaining two cases, this source seems unlikely, and transmission might have occurred indirectly from contaminated sources, such as wastewater and home-grown vegetables. Further studies are needed to investigate if these latter sources may be more common than previously thought or may play a role in relation to specific HEV subtypes and/or only in rare and exceptional circumstances.

## 1. Introduction

Hepatitis E Virus (HEV) is a member of the Hepeviridae family, whose members infect animal species and/or humans [1]. Eight genotypes have been recognized for this virus (HEV-1 to HEV-8) [2]. While HEV-1 and HEV-2 infect exclusively humans, HEV-3 and HEV-4 are mainly animal viruses that also show zoonotic transmission to humans (but not direct human-to-human transmission through close contact; human-to-human transmission has been described in specific circumstances, such as blood transfusion from an infected donor or indirect exposure through contaminated water). The other genotypes (HEV-5 to HEV-8) have been described in several animal species but not in humans, with the only exception of a single reported case of infection by HEV-7, likely caused by particular and exceptional circumstances [3].

HEV-1 and HEV-2 are mainly transmitted by contaminated water and are responsible for recurrent waterborne epidemics in Asia and Africa; in Western countries these genotypes are uncommon and can be detected in travelers returning from endemic areas.

In contrast, HEV-3 and HEV-4 infections are believed to be mainly food-borne and in the great majority of cases linked to the consumption of raw or undercooked pork products and wild boar meat from infected animals [4,5]. However, providing direct evidence of transmission from food has proved to be challenging, both because of the long incubation (so that at the onset of symptoms the suspected food is no longer available for sampling in most cases) and because of technical difficulties in obtaining HEV sequences from food for comparison with sequences from the cases; in fact, this evidence has been reported only rarely [6].

In the last two decades Europe has experienced an increase in autochthonous hepatitis E cases, unrelated to travel to endemic countries; autochthonous cases are caused by HEV-3 strains [7]. HEV-3 is classified in several subtypes according to the criteria established by an international consensus in 2016, updated in 2020 [2,8]. The criteria included phylogenetic analysis of sequences with established subtype reference sequences and p-distance analysis (intra-subtype distances are usually <0.125 but can range up to 0.134 in subtype 3f) [8]. In the last revision of the criteria, it was also suggested that sequences may be “grouped together into one subtype where there are no gaps in the distribution of distances and the group shares a common branch” in a phylogenetic tree, without imposing an arbitrary cut-off [8]. The recognized HEV-3 subtypes have progressively increased, and to date, the official list includes 14 subtypes (3a to 3m, plus the 3ra infecting rabbit).

Different European regions tend to show different distribution of HEV-3 subtypes in humans, as shown by a report of data collected in the same year from the national HEV surveillance systems: in 2014, the predominant HEV-3 subtypes were 3c in England and Wales; 3f in France; 3c in Germany; 3f, 3e and 3c in Italy; 3c in the Netherlands; and 3f in Spain [7]. A recent study in Hungary reported subtype 3a to be the most common, followed by 3c, 3f and 3e; other rare subtypes (3g, 3h, 3i, 3m and 3ra) were also detected, overall accounting for less than 10% of the cases [9].

In Italy most hepatitis E cases have so far been reported to be caused by 3f, 3c and 3e subtypes, rarely by 3a or other still undefined subtypes [10,11]. The most recent of such studies focused on cases reported in Lazio and Abruzzo (two regions of Central Italy) and showed that the relative proportions of 3f, 3c and 3e subtypes among hepatitis E cases remained quite stable throughout the observation period (2015 to 2023), with the 3f subtype being the most abundant and annually detected in half of the cases [11]. Central Italy had been previously shown to be an area of high HEV circulation by anti-HEV seroprevalence studies [12,13,14].

Studies in Italy in several animal species (swine, wild boar, sheep, goat) show that the detected HEV-3 subtypes and their relative proportions may vary according to the animal host, the geographical region and the year of sampling. A summary of such studies in Italy follows.

In farmed pigs, sampling in the Piedmont region (Northern Italy) earlier than 2016 reported detection of 3f, 3e and 3c [15]; in Northern Italy, the novel 3l subtype was also identified [16]. A study carried out in 2020–2022 in pig farms from eight European countries, including Italy, confirmed that 3f, 3e and 3c are the most frequently detected subtypes in farmed pigs in Italy; however, 3l-like and unclassified potential new subtypes were also occasionally detected [17]. A recent study (sampling 2022–2023) in home-slaughtered domestic pigs in the Ascoli Piceno province (Marche region, Central Italy) detected exclusively the 3c subtype, i.e., the same subtype also detected in wild boars sampled in 2020–2021 in the same province (see below) [18]. A study in Sicily in 2021–2022 in slaughtered pigs detected the 3c subtype in 18/24 (75%) animals [19]. To our knowledge, no study has reported the detection of the 3i subtype in pigs in Italy.

In contrast to pigs, a far greater diversity of subtypes has been observed in wild boars in Italy, including strains belonging to as-yet unassigned subtypes; the variable distribution of subtypes in wild boar in different studies is most likely related to variation in geographical and temporal sampling.

In Northern Italy 3f, 3e, 3a and 3c subtypes have been observed in wild boars between 2012 and 2019, and, less frequently, in the Veneto region, unclassified potential novel subtypes [20,21,22,23,24].

In Central Italy, wild boar samplings in Abruzzo between 2015 and 2019 showed that 3c was the most abundant subtype, but 3f and 3e were also detected [25,26,27,28]); wild boar samplings in Marche (Ascoli Piceno province, bordering Abruzzo) between 2019 and 2021 detected exclusively the 3c subtype [29,30]; wild boar sampling in Lazio (Viterbo province) between 2011 and 2014 and between 2016 and 2017 showed unassigned subtypes in roughly one half samples, whereas 3c and 3f were the most frequent subtypes in the remaining one half, with 3a and 3l also detected but just in a few samples [31,32]. A further study of wild boar samples collected in Umbria and Marche during the hunting seasons 2016–2020 detected 3f, 3e and 3c subtypes in 7/15 HEV positive samples; however, the strains detected in the remaining 8/15 samples were related to a strain from South Italy previously classified as 3i [33], formed an independent branch in a phylogenetic tree and were proposed to be members of a novel subtype tentatively termed 3n [34].

In Southern Italy, the 3c subtype is the most frequently detected in wild boars. Sampling in Campania between 2015 and 2016 detected the 3c (although the uncommon 3j-like was also reported [25]. Sampling in Apulia and Basilicata in 2021 detected the 3c [35]. Sampling in Calabria between 2019 and 2020 detected the 3c subtype in 19/23 (83%) animals [36]. Although data clearly show that the 3c subtype is predominant in South Italy wild boars, no known subtype could be assigned to the remaining 4/23 animals in the Calabria study, and, in addition, unknown or unusual subtypes, including subtype 3i, were also described in another study [33]. Subtyping of the HEV strains detected in other animal species showed the 3c subtype in both sheep and goats in Abruzzo [37,38].

The present study documents for the first time the detection of the 3i subtype in human hepatitis E cases in Italy. The cases were resident in a small geographical area in Umbria, Central Italy. According to the hypothesis of transmission from the animal reservoir, possibly including indirect transmission from contaminated sources, three of the five strains proved to be highly related to some of the strains observed in wild boars sampled in the same geographical area in two previous studies covering hunting seasons 2016–2017, 2017–2018 and 2018–2019 [34] and hunting season 2021–2022 [39].

## 2. Materials and Methods

### 2.1. Samples

In the frame of the national virological surveillance of hepatitis E, between 2019 and 2023, the Microbiology Laboratory of the Perugia Hospital (Umbria, Italy) sent serum samples from 10 overall hospitalized cases of acute hepatitis E to the Viral Hepatitis and Oncovirus and Retrovirus Diseases Unit of the Department of Infectious Diseases, Istituto Superiore di Sanità (the National Institute of Health). Upon receipt, they were stored at −80 °C until RNA extraction by the QIAmp MinElute Virus Spin kit (QIAGEN, Hilden, Germany). During hospitalization, patients were interviewed through a standard questionnaire including questions about possible exposure to risk factors for HEV infection in the eight weeks preceding the onset of symptoms.

Wild boar liver samples positive for HEV RNA (n = 41), available for the present study, were collected within a previous study performed in Umbria during hunting season 2021–2022 in the framework of regional HEV surveillance in this animal reservoir [39].

### 2.2. Viral Characterization by Sanger Sequencing

Routine virological surveillance of human hepatitis E is carried out through viral characterization by Sanger sequencing of a HEV genome fragment amplified by nested PCR from the ORF2 region, leading to a 493 nt sequence, according to the ECDC protocol made available by the RIVM and previously described [40]. The HEV sequences are then genotyped and sub-genotyped by phylogenetic analysis with standard reference sequences and previously detected strains [11].

HEV in the 41 wild boar liver samples had been previously characterized by nested PCR followed by Sanger sequencing, yielding a 410 nt partial ORF2 sequence [39] (GenBank Accession Number OQ349517-OQ349557). To improve understanding of circulating strains, a selection of twelve samples yielding sequences previously described as ‘3uc’ or ‘unclassified’ in the original study were subjected to amplification of a 1117 nt fragment of the ORF 2 region. Briefly, the RT-PCR was carried out in a 25 μL reaction volume, using the Superscript IV One-Step RT-PCR System (Thermo Fisher Scientific, Waltham, MA, USA), 5 μL of sample RNA and 0.4 μM of forward primer (JVHEVF: 5′-GGTGGTTTCTGGGGTGAC-3′) [41] and reverse primer (HE040: 5′-CCCTTRTCCTGCTGAGCRTTCTC-3′) [42] with the following conditions: reverse transcription at 50 °C for 20 min, inactivation for 2 min at 98 °C, followed by 40 cycles of 98 °C for 10 s, 60 °C for 20 s, 72 °C for 1 min and a final step of 72 °C for 10 min. Two μL of the first PCR product was used as template in a hemi-nested reaction, that used Platinum SuperFi II PCR mastermix (Thermo Fisher Scientific), 0.4 μM of primer JVHEVF and nested primer HE041 (5′-TTMACWGTCRGCTCGCCATTGGC-3′) [42] and the following thermal profile: 1 min at 98 °C, 45 cycles of 98 °C for 10 s, 60 °C for 20 s, 72 °C for 1 min, and a final step of 72 °C for 10 min. PCR products were purified using the GRS PCR & Gel Purification Kit (GRiSP, Porto, Portugal), and samples were sequenced on both strands through a commercial service (EuroFins Genomics, Ebersberg (Bayern), Germany).

### 2.3. Viral Characterization by Next-Generation Sequencing

In the present study, further virological characterization of five human strains with unusual HEV-3 subtypes was attempted by a full-genome Next-Generation Sequencing approach, described in detail in a previously submitted manuscript currently under review and available as a not-peer-reviewed pre-print at this stage [28]. Briefly, each RNA extract was reverse transcribed by the Ion Torrent™ NGS Reverse Transcription Kit (Thermo Fisher Scientific, Waltham, MA, USA). A library was prepared from each sample by the Ion AmpliSeq™ Library Kit Plus (Thermo Fisher Scientific) with custom primer pools designed by Thermo Fisher Scientific and IonCode™ Barcode Adapters 1-96 Kit (Thermo Fisher Scientific), according to the manufacturer’s instructions. The quality, average size and concentration of each library were assessed by an Agilent TapeStation, software version 5.1 (Agilent Technologies, Santa Clara, California, USA). After pooling of the libraries and a step of emulsion PCR, the samples were sequenced on an Ion GeneStudio S5 System according to the manufacturer’s instructions (Thermo Fisher Scientific). Bioinformatics processing and analysis of the output reads were carried out by tools available on the Torrent suite software version 5.18.1 (Thermo Fisher Scientific). The procedure was successful for three of the five strains. Almost the whole-genome sequences (about 7 Kb) of the three strains and the short Sanger sequences of the remaining two strains were deposited in GenBank under Accession Numbers PX682538, PX682539, PX682540, PX725478 and PX725488.

### 2.4. Sequence Analysis

Sequence analysis was carried out by MEGA 12 [43]. Sequences were aligned by the Muscle tool in MEGA 12. To optimize the alignment, it was inspected and manually edited, wherever needed. The alignment included recommended HEV-3 subtype reference sequences [8] as well as both HEV sequences that had shown high nucleotide identity with case sequences by BLAST search in the NCBI database [44] and HEV sequences previously reported from the same geographical area. Phylogenetic analysis included preliminary evaluation of the best evolutionary model for the dataset under investigation by the Model tool, followed by phylogenetic tree construction by the Maximum Likelihood approach, with the best evolutionary model and the bootstrap set to 1000.

Analysis of the p-distances was carried out by the Distance tool in MEGA 12. A dot plot representation of the distances was done by Canva [45].

## 3. Results

### 3.1. Identification of HEV Subtypes Not Previously Detected in Humans in Italy

In the frame of national virological surveillance of hepatitis E, the Viral Hepatitis and Oncovirus and Retrovirus Diseases Unit of the Department of Infectious Diseases at Istituto Superiore di Sanità (the National Institute of Health) receives serum samples from suspected or confirmed hepatitis E cases for virological surveillance purposes, including viral characterization by sequencing of an ORF2 fragment (493 nt) of the HEV genome. Overall, 119 samples were received from throughout Italy for HEV testing in the period 2019–2023, and 54 of them (45%) were confirmed to be hepatitis E cases. Ten of these cases had been hospitalized in the Perugia Hospital in 2019, 2022 and 2023. They were resident in the Umbria region (nine cases) and in the nearby province of Viterbo (Lazio region), bordering Umbria (one case). Among them, five cases proved to be caused by 3f (n = 3), 3e (n = 1) and 3c (n = 1) subtypes, as usually observed in hepatitis E cases in Central Italy [11]; however, the strains observed in the remaining five cases clustered in the clade 3c-h-i-l-m but could not be clearly classified into any of those officially recognized subtypes. The remaining 44/54 HEV positive cases from throughout Italy led to an ORF2 sequence in 33 cases, and the following genotypes/subtypes were detected: 3f (n = 15), 3e (n = 13), 3c (n = 4) and 1g (n = 1) (this latter from a Pakistani patient).

### 3.2. Characterization and Analysis of the HEV Strains with Unusual Subtype

**Figure 1:**
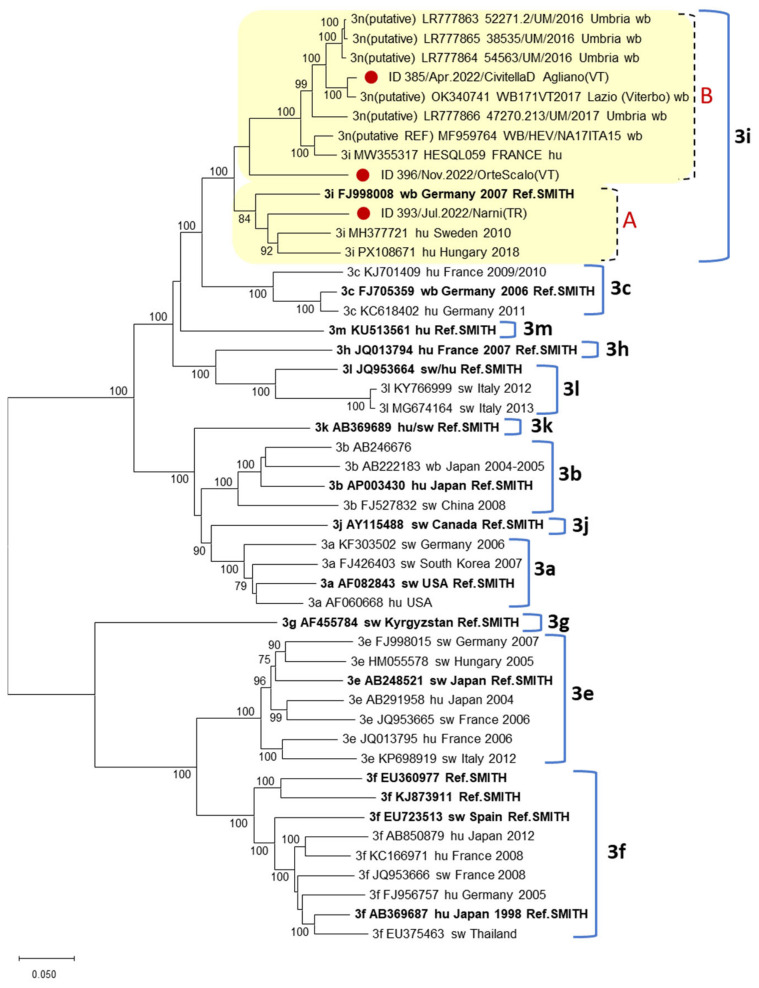
Phylogenetic tree including whole-genome HEV sequences (a) from three hepatitis E cases (red circles), (b) established by an international consensus to be used as reference sequences for accepted subtypes, 3a to 3m (in bold) [2,8], (c) shown, upon BLAST search, to have high identity (>86%) with sequences from the cases [44] and (d) previously described animal sequences sampled in the Italian area in which the three cases occurred. The following abbreviations indicate the host from which the sequence was obtained: hu, human; sw, swine; wb, wild boar.

To better characterize those strains with an undetermined HEV-3 subtype, a Next-Generation Sequencing approach was attempted to obtain their whole-genome sequences. The approach was successful for three of the five sera: a nearly complete HEV genome sequence could be obtained (coverage: 97.2–98.07%; sequence length: 7059–7065 nt) from hepatitis E cases (ID385, ID393 and ID396). Then, the three nearly complete HEV genome sequences were subjected to phylogenetic analysis. A proper sequence dataset was built including (a) the three sequences to be analyzed, (b) the whole-genome reference sequences established by an international consensus to be used as reference for accepted HEV-3 subtypes [2,8], (c) whole-genome sequences that had shown high identity (>86%) with our nearly complete HEV sequences upon BLAST search [44] and (d) previously described whole-genome animal sequences sampled in the Italian area in which the five cases occurred. Figure 1 shows the resulting phylogenetic tree.

As expected, due to the larger amount of genetic information provided by whole-genome sequences than short Sanger sequences, most nodes in the tree were supported by statistically significant bootstrap values. The sequences from the three cases cluster in a branch together with 3i references, as well as with sequences retrieved upon BLAST search and/or sampled from roughly the same geographical area in which the cases occurred. These latter sequences were previously proposed to represent a novel subtype, tentatively termed 3n, that is not yet included in the last available update of officially recognized HEV-3 subtypes, whose publication preceded the proposal of the 3n subtype [8,34]. The branch is split into two well-distinct and statistically supported clusters (bootstrap value = 100): one cluster, labeled “A” in Figure 1, includes the ID393 sequence and the 3i references, whereas the other includes the ID385 and ID396 sequences together with sequences of the proposed 3n subtype (cluster “B” in Figure 1).

**Figure 2:**
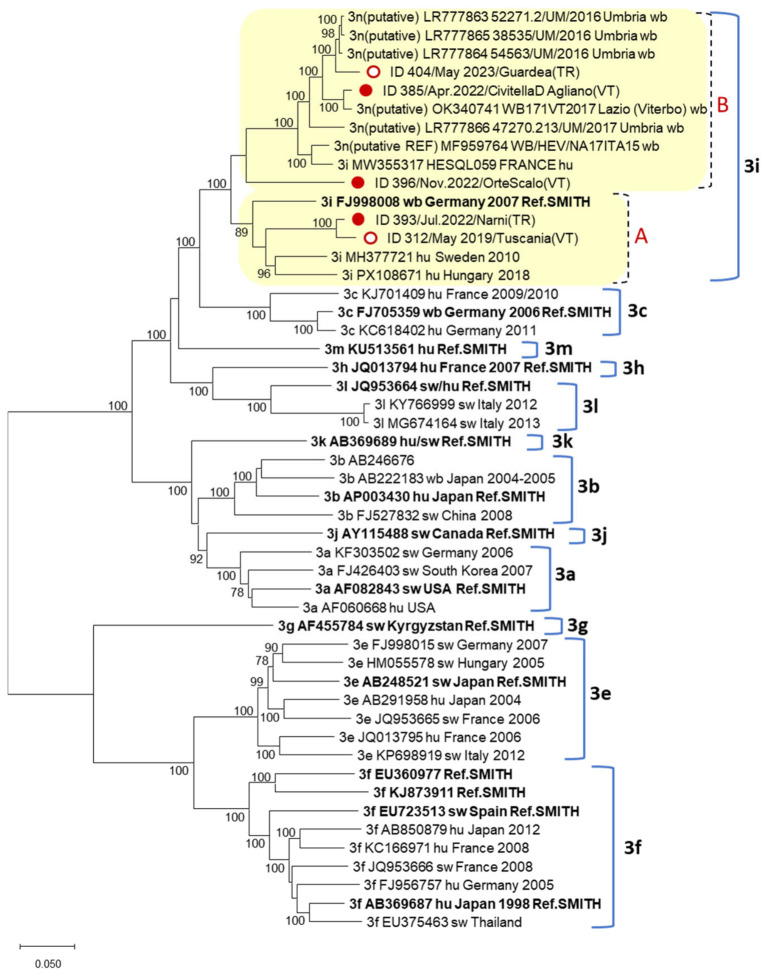
Phylogenetic tree including, in addition to the whole-genome sequences used for the tree in Figure 1, also the short Sanger sequences available from two additional cases (empty red circles).

Then, the phylogenetic analysis was extended to the two other strains with unusual subtypes (ID312 and ID404) by including their available short Sanger sequences (493 nt) in the whole-genome dataset. The parameters of the phylogenetic analysis were set to tolerate any alignment positions containing up to 4% gaps (i.e., no more than two gaps at any position of the aligned 50 sequences): this resulted in all the sequences (long and short) being compared through the shared 493 nt region, whereas the long sequences only being analyzed throughout about 6100 positions. Figure 2 shows the resulting phylogenetic tree: the two short sequences also clustered in the branch containing 3i and putative 3n sequences; in particular, ID312 clustered with ID393 (cluster “A” in Figure 2) and ID404 clustered with ID385 (cluster “B” in Figure 2).

A topologically similar phylogenetic tree was obtained by limiting analysis exclusively to the short 493 nt region shared by long and short sequences; however, as expected, several nodes showed bootstrap values < 70.

### 3.3. p-Distance Analysis

In most known HEV-3 subtypes, whole-genome p-distance values between members of the same subtype are lower than 0.125, with the only exception of the 3f subtype with values up to 0.134 [8]: so, in general, a distance < 0.125 suggests (although does not definitively prove) that the two compared sequences are members of the same subtype.

To further evaluate if members of the two phylogenetic clusters in the 3i branch (Figure 1, A and B clusters) may indeed be considered as two distinct subtypes, the nucleotide p-distances between all whole-genome sequence pairs of the 3i branch were calculated. All the p-distance values were ≤0.125, suggesting all sequences belong to the same subtype.

However, in the last revision of the criteria for subtype assignment, it has also been suggested that instead of imposing an arbitrary cut-off on a continuous distribution of distances, sequences may be grouped together into one subtype where there are no gaps in the distribution of distances and the group shares a common branch [8]. This means, conversely, that two phylogenetic groups can be assigned to two different subtypes if the intra-group distances are separated by a gap from the inter-group distances.

**Figure 3:**
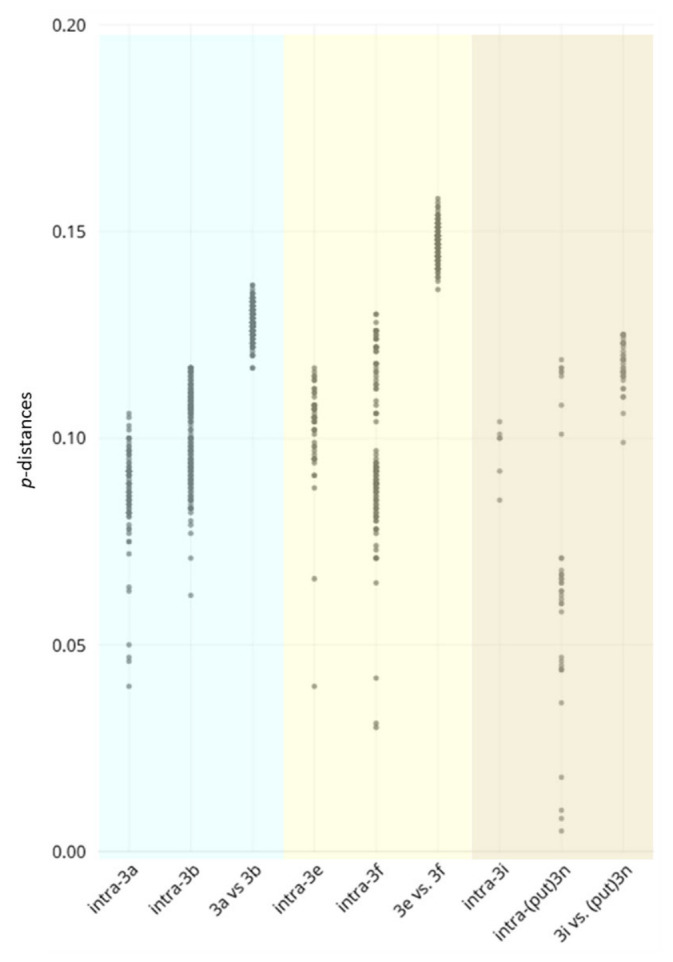
Dot-plot distribution of intra- and inter-group p-distances obtained from HEV whole-genome sequences classified into different subtypes: 3a vs. 3b (light blue shaded), 3e vs. 3f subtype (yellow shaded) and 3i vs. (put = putative) 3n subtype (light orange shaded).

Figure 3 shows that, although the 3i and putative 3n sequences clustered in two separate branches in the tree shown in Figure 1, there is no gap in the distribution of distances: the intra-3i and intra-3n values are not at all separated from the inter-group distances that, actually, overlap intra-group distances, suggesting all sequences are members of the same subtype.

Figure 3 also shows, as comparison and control, the results of the same p-distance analysis on whole-genome sequences for 3a vs. 3b strains and 3e vs. 3f strains: for both pairs, the sequences cannot be grouped into one subtype because the intra-group 3a and 3b, as well as 3e and 3f distances, are separated from the inter-group distances, i.e., in these cases, there is a gap in the distribution of p-distances, as expected when the compared strains belong to different subtypes.

Thus, although phylogenetic analysis segregates whole-genome HEV sequences from cases ID393, ID385 and ID396 into two separate clusters, at present the distance analysis allows us to conclude that they are members of the 3i subtype. Nevertheless, the potential existence of a distinct evolutionary lineage within the 3i subtype that might be evolving into a distinct subtype deserves further investigation as more whole-genome sequences become available.

### 3.4. Analysis of Information Available from the Five Cases

The first case with an unusual subtype was detected in May 2019; it appeared to be an isolated case as no further cases with an unusual subtype were observed in the same year, as well as in 2020 and 2021. However, four further cases were detected starting three years later: in April, July and November 2022 and in May 2023. Table 1 summarizes the available laboratory results and the investigation of risk factors.

All five cases were Italian, median age 59 years (range: 50–67 years); four were males. All cases were anti-HEV IgM positive by ELISA assay and showed HEV RNA ≥ 10^4^ copies/mL.

Three cases (ID385, ID396, ID404) reported the consumption of raw/undercooked pig products and/or wild boar products during the eight weeks preceding symptom onset; one case (ID312) reported consumption of shellfish but also referred to be a wastewater treatment operator and, finally, one case (ID393) reported exclusively consumption of home-grown vegetables (vegetables grown in his own garden), absolutely denying any other investigated risk factor. No further information was available regarding the irrigation sources used for home-grown vegetables (e.g., individual well, municipal water source, etc.), nor about the potential access of animal reservoirs to the vegetable cultivation area. No cases referred with a history of travel abroad. The towns of residence of the five cases were in a restricted area of Central Italy: the distance from each other was between 20 km and 70 km.

### 3.5. Comparison with HEV Sequences Collected from Wild Boars in Umbria in 2021–2022

In the framework of a surveillance study on the wild boar reservoir [39], the HEV sequences (ORF2 region of the viral genome) from 41 wild boars sampled in Umbria were available to be compared with the sequences from the five hepatitis E cases. Wild boars had been sampled in hunting season 2021–2022 (November 2021 to February 2022), i.e., almost three years after the onset of the first case (ID312, onset in May 2019) and 2–18 months before the onset of the four later cases (ID385, ID393, ID396, ID404, onset in April, July and November 2022 and May 2023, respectively). The size of all but one wild boar HEV sequences was 410 nt. A larger 1085 nt sequence (Acc. No. PX757462) was available from one sample (wbUM098), as a result of a successful attempt to amplify a larger fragment from a selection of twelve samples, using different primers (see Section 2 for details).

The 41 HEV sequences from wild boars were included in the dataset used to build the phylogenetic tree shown in Figure 2. Then, the new dataset underwent phylogenetic analysis, limited to the 410 nt region shared by all sequences.

**Figure 4:**
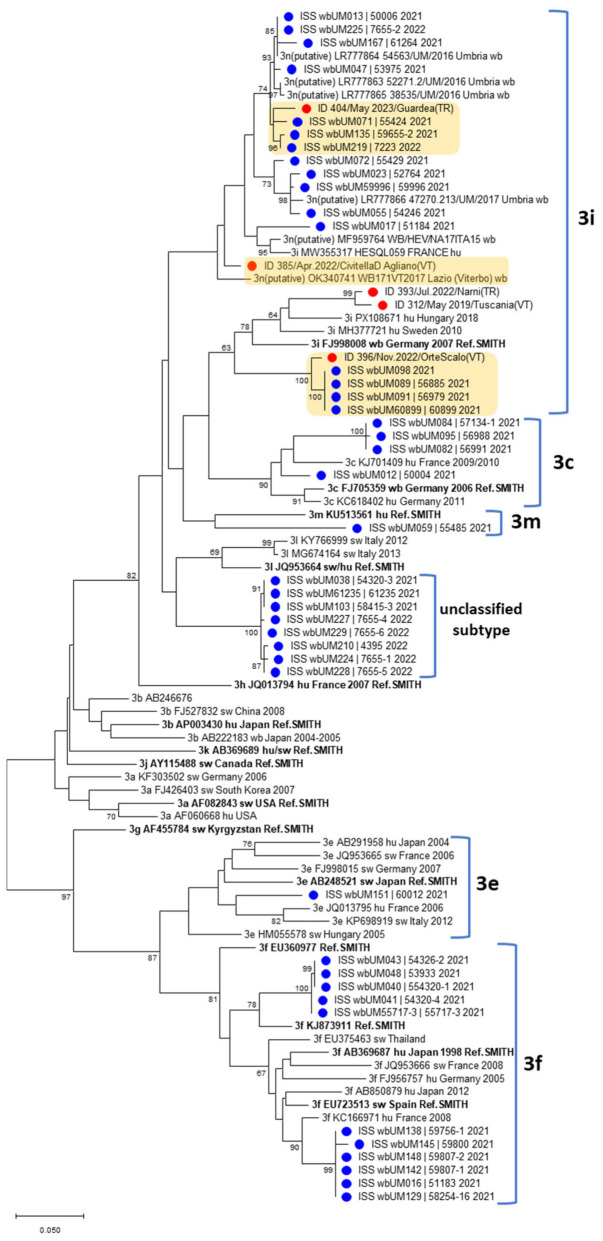
Phylogenetic tree based on analysis of a 369 nt overlapping region shared by human and wild boar sequences, including (a) HEV sequences from hepatitis E cases (n = 5, red circles), (b) sequences from wild boars of the present study (n = 41, blue circles), (c) reference sequences (subtype 3a to 3m, see also caption of Figure 1), (d) sequences shown to have high identity (>86%) with sequences from the cases upon BLAST search [44] and (e) previously described animal sequences sampled in the Italian area in which the cases occurred.

Figure 4 reports the resulting phylogenetic tree, which shows several nodes with non-significant bootstrap values due to the too short analyzed region.

The sequences from wild boars could be assigned to the following subtypes: 3i (n = 16), 3c (n = 4), 3m (n = 1), unclassified 3 subtype (n = 8), 3e (n = 1), 3f (n = 11). Overall, 16/41 sequences (39%) from wild boars showed the same 3i subtype as the sequences from the five hepatitis E cases.

In addition, Figure 4 shows that the sequences from three of the five cases clustered with specific sequences from wild boars: in detail, ID404 (onset May 2023) clustered with three wild boar sequences (wbUM071, sampled in November 2021, wbUM135, sampled in December 2021 and wbUM219, sampled in February 2022); ID396 (onset Nov. 2022) clustered with four sequences from wild boars sampled in December 2021 (wbUM089, wbUM60899, wbUM091 and wbUM098); ID385 (onset Apr. 2022) was related to the sequence Acc. No. OK340741 sampled in 2017 in the same province: although the bootstrap was not significant in this analysis, this was most likely due to the tree being based on 410 nt, as the corresponding whole-genome sequences showed high identity (98.2% over 7002 nt) and clustered together (Figure 1). The remaining two human HEV sequences, ID312 and ID393, clustered together, but with no other human or animal HEV sequences in the tree.

As reported above, a larger sequence (1085 nt) was available from a single wild boar sample (wbUM098). To exploit its entire genetic information, this 1085 nt sequence was analyzed individually: it was included in a dataset retaining only the whole-genome sequences (all short sequences were removed), and the phylogenetic analysis was repeated to analyze the 1085 nt region shared by all sequences. Supplementary Figure S1 shows the resulting phylogenetic tree, which confirms the clustering of ID396 with the wbUM098 sequence from wild boar, as already observed with the short wbUM098 sequence.

Table 2 reports, for each of the above three human/wild boar phylogenetic clusters highlighted in Figure 4, the number of nucleotide differences and the percent nucleotide identity between human and wild boar HEV sequences, as well as the distance between the residences of cases and wild boar sampling sites.

The size of the compared regions ranged from 369 to 410 nt, according to the actual overlapping region between the human whole-genome and wild boar short HEV sequences. Exception was the compared region of ID385 vs. OK340741: this latter was a whole-genome sequence too, so the results are reported for both the long (7002 nt) and the short region (410 nt); the identity over the 410 nt region (94.9%) proved to be quite lower than the identity over the 7002 nt region (98.2%).

Each HEV sequence from humans exhibits a high degree of nucleotide identity (94.9 to 98.2%) with the wild boar sequences of its own phylogenetic cluster, supporting the hypothesis of a common evolutionary origin of human and wild boar sequences in each cluster. Interestingly, ID396 shares a characteristic ATG codon (in place of ACG) with the four wild boar sequences forming the cluster: the ATG is responsible for coding a Methionine in place of the Threonine coded by the other 3i strains of the dataset and, thus, results in an “amino acid mark” specific of these strains, further supporting the hypothesis of common evolutionary origin of members of this cluster.

Overall, the phylogenetic analysis and the sequence identity values provide evidence that ID385 is related to a wild boar strain sampled five years earlier, 28 km apart; ID396 is related to wild boar strains sampled one year earlier, 27 km and 128 km apart; and ID404 is related to wild boar strains sampled 1–1.5 years earlier, 19 km and 60 km apart.

## 4. Discussion

To our knowledge, this is the first report documenting hepatitis E cases caused by 3i subtype in Italy, where 3f, 3c, 3e and, rarely, 3a are usually detected in humans. In fact, 3i-like strains related to those described in the present investigation had been previously reported in Italy exclusively from wild boars. The present study also reports that related 3i-like strains were circulating in wild boars sampled in the same geographical area as the cases and during a time period overlapping the onset date of the cases, providing evidence of a geographical and temporal match between animal and human infections by 3i strains.

Whole-genome HEV sequencing, successful in three of five cases, allowed for more reliable phylogenetic analysis than conventional Sanger sequencing, with most nodes in the phylogenetic tree supported by statistically significant bootstrap values. Whenever possible, whole-genome sequencing should be carried out for the best reconstruction of viral circulation in humans and animals.

Phylogenetic analysis of the three whole-genome and the two short sequences from the five cases showed that the strains clustered in a well-defined and statistically supported branch, which included 3i references as well as other sequences downloaded from databases. The branch was split into two well-separated and statistically supported sub-clusters: the strains from two cases clustered with 3i references, the strains from the three other cases clustered with strains from wild boars previously proposed as members of a novel subtype, tentatively termed 3n, still not officially recognized yet. However, based on the current HEV subtype definition that includes both phylogenetic and p-distance analysis, the strains of the cases appear to be members of the same subtype, 3i, even though they segregate into two main sub-clusters in the phylogenetic tree [8]. Whether one of the two sub-clusters may represent a distinct evolutionary lineage within the 3i subtype, with ongoing evolution into a distinct subtype, deserves further investigation in the future, when additional whole-genome sequences become available.

The results of several studies in pigs and wild boars in the last decade in North, Central and South Italy (see Section 1) have shown that, to date, 3i-like subtypes have been detected as a rare occurrence exclusively in wild boars in Central and South Italy [33,34]. In particular, in Central Italy, 3i-like strains have been detected in wild boars both in hunting season 2016–2017 [33,34] and in 2021–2022 [39], suggesting continuous local circulation in wild boars over time. The five hepatitis E cases of the present study occurred in 2019–2023 in Central Italy, thus with space-time overlap with the sampling area and sampling date of wild boars that harbored 3i-like strains. As the 3i subtype had never been detected previously in humans in Italy, it can be hypothesized that local circulation of 3i-like strains in wild boars may have increased in recent years, thereby increasing their chance for local transmission to humans.

Although the HEV sequences from three cases showed high identity to sequences from wild boars, they were not identical, raising the question of whether wild boars were actually the ultimate source of human HEV infections. However, the observed sequence divergence is not unexpected: in fact, the observed wild boar strains cannot be considered as the exact strains that gave rise to infection in individual cases, but rather they just represent a sample of the HEV strains overall circulating in wild boar populations (and, possibly, in home raised pigs) in this area in 2021–2022 (i.e., two years following onset of the 2019 case and one-year preceding onset of the 2022–2023 cases). Thus, the observed sequence divergence between human and animal strains can be explained in terms of both genetic variations occurring and accumulating over time during animal-to-animal transmission and, additionally, as a likely consequence of the expected virus adaptation to the human host.

According to the reported risk factors, HEV infection in three of the five cases had most likely occurred through consumption of raw/undercooked products (such as cured sausages) from wild boars and/or pigs. Consumption of cured products from pigs plausibly involves also consumption of products from home-raised animals, due to the local habit of free-range pig farming for familial consumption, widely diffused in this area and, more in general, in Central Italy. This habit allows for interaction of pigs with the wild boar population, giving a chance for transmission of wild boar strains to humans, also indirectly, through pigs infected by wild boars. Unfortunately, in the present study, no consumed food could be investigated, so direct evidence for transmission by demonstration of identical or almost identical HEV sequences in patients and food could not be provided. This is a common problem mainly due to the long incubation period preceding the onset of symptoms, so that in most cases the suspected food is no longer available for investigation when symptoms occur; additionally, even when food is available, HEV detection and sequencing from food matrices are frequently unsuccessful, being more difficult than from patient serum. Indeed, direct demonstration of food transmission has been reported only in rare cases [6].

It is worth noting that two of the five cases denied any consumption of wild boar or pig products. Although recall bias and unaware consumption (e.g., due to presence in food preparations) cannot be completely excluded, other possible sources of infection in these two patients cannot be excluded either, such as contact with untreated wastewater in one case and consumption of contaminated home-grown vegetables in the other case, according to information provided by these two patients. In particular, transmission through contaminated home-grown vegetables might be more significant than is generally expected, due to the uncontrolled increase in the wild boar population in recent years, with its impact in terms of environmental contamination (shedding of HEV on soil, leaching of virus to water sources), potential virus transmission to free-ranging home-raised pigs and possible direct contamination of crops/vegetables in accessible areas.

## 5. Conclusions

In summary, the following observations provide circumstantial evidence that the observed five hepatitis E cases caused by 3i strains were indeed caused by HEV spillover either directly from wild boars/home-raised pigs or indirectly from contaminated sources: (a) the relatively small geographical area in which the cases occurred, suggesting local transmission, (b) the finding that HEV sequences from three cases showed high identity versus HEV sequences specifically found in wild boars circulating in the same area of residence of the cases and (c) the risk factors resulting from interview of the cases.

At least in three of the five cases, HEV infection was most likely acquired through consumption of raw/undercooked products from local wild boars or home-raised pigs. However, in the remaining two cases, this source of infection seems to be unlikely and, according to the collected information, HEV transmission might have occurred indirectly from contaminated sources such as untreated wastewater and home-grown vegetables. Further studies are needed to investigate if these latter sources may be more common than previously thought or may play a role in relation to specific HEV subtypes and/or only in rare and exceptional circumstances.

## Figures and Tables

**Table 1:** Laboratory data and risk factors of the five cases.

	ID312	ID385	ID393	ID396	ID404
Laboratory data					
Anti-HEV IgM (ELISA)	+	+	+	+	+
HEV RNA (copies/mL)	>10^5^	10^4^	>10^5^	>10^5^	10^4^
AST/GOT (U/L)	3061	1919	963	1000	549
ALT/GPT (U/L)	1565	2687	549	1000	1097
Direct Bilirubine (mg/dL)	1.33	2.2	8.87	4.5	0.5
Total Bilirubine (mg/dL)	4.98	2.6	14.7	5	1.4
Gamma-Glutamyl Transpeptidase (GGT) (U/L)	3	110	71	200	n.a.
Alkaline phosphatase (ALP) (U/L)	n.a.	282	256	500	n.a.
Risk factors					
Consumption of raw or undercooked meat from pig, wild boar, deer or other wild game meat	-	-	-	-	+
Consumption of cured (uncooked) pig sausages	-	+	-	+	+
Consumption of cured (uncooked) wild boar sausages	-	+	-	-	+
Consumption of pig/wild boar cured (uncooked) liver sausages	-	-	-	-	-
Consumption of home-grown vegetables	-	+	+	-	-
Consumption of shellfish	+	-	-	-	-
Travel abroad	-	-	-	-	-

n.a. = not available.

**Table 2:** Comparison of the strains of the three clusters highlighted in Figure 4.

Human		Wild Boar		nt Differences vs. Total nt Compared	% nt Identity	Distance Between Residence of Case and Wild Boar Sampling Site
Strain	Onset Date	Strain	Sampling Date			
ID385	April 2022	OK340741	2017	123/7002 21/410	98.2 94.9	28 km
ID396 *	November 2022	wbUM089 *	December 2021	9/410	97.8	27 km
		wbUM091 *	December 2021	9/410	97.8	128 km
		wbUM098 *	December 2021	34/1085 9/410	96.9 97.8	128 km
		wbUM60899 *	December 2021	9/410	97.8	27 km
ID404	May 2023	wbUM071	November 2021	11/369	97.0	19 km
		wbUM135	December 2021	12/369	96.7	60 km
		wbUM219	February 2022	11/369	97.0	n.a.

* These human and wild boar strains share an ATG codon in place of ACG (T in 2nd position) that is responsible for coding a Methionine in place of Threonine in the ORF2. n.a.: not available.

## Data Availability

The HEV sequences from the five cases were deposited in GenBank under Accession Numbers PX682538, PX682539, PX682540, PX725478 and PX725488. The HEV sequences from wild boars sampled in the same areas as the cases were described in a previous study and deposited in GenBank under Accession Number OQ349517-OQ349557 [39], except for one new sequence, which is longer than the one previously obtained from the same animal (Accession Number PX757462).

## Supplementary Materials

The following supporting information can be downloaded at https://www.mdpi.com/article/10.3390/v18070709/s1. Figure S1: Phylogenetic tree resulting from individual analysis of the 1085 nt version of the wild boar sequence from wbUM098. The tree confirms the clustering of ID396 with the wbUM098 strain from wild boar, as already observed with the short sequence version of wbUM098 (see Figure 4).
