# Supplementary figures and images for: First Identification of HEV Subtype 3i in Human Hepatitis E Cases in Central Italy
Source: Viruses. 2026 Jun 27;18(7):709. doi: 10.3390/v18070709 (PMC13431625; doi:10.3390/v18070709)

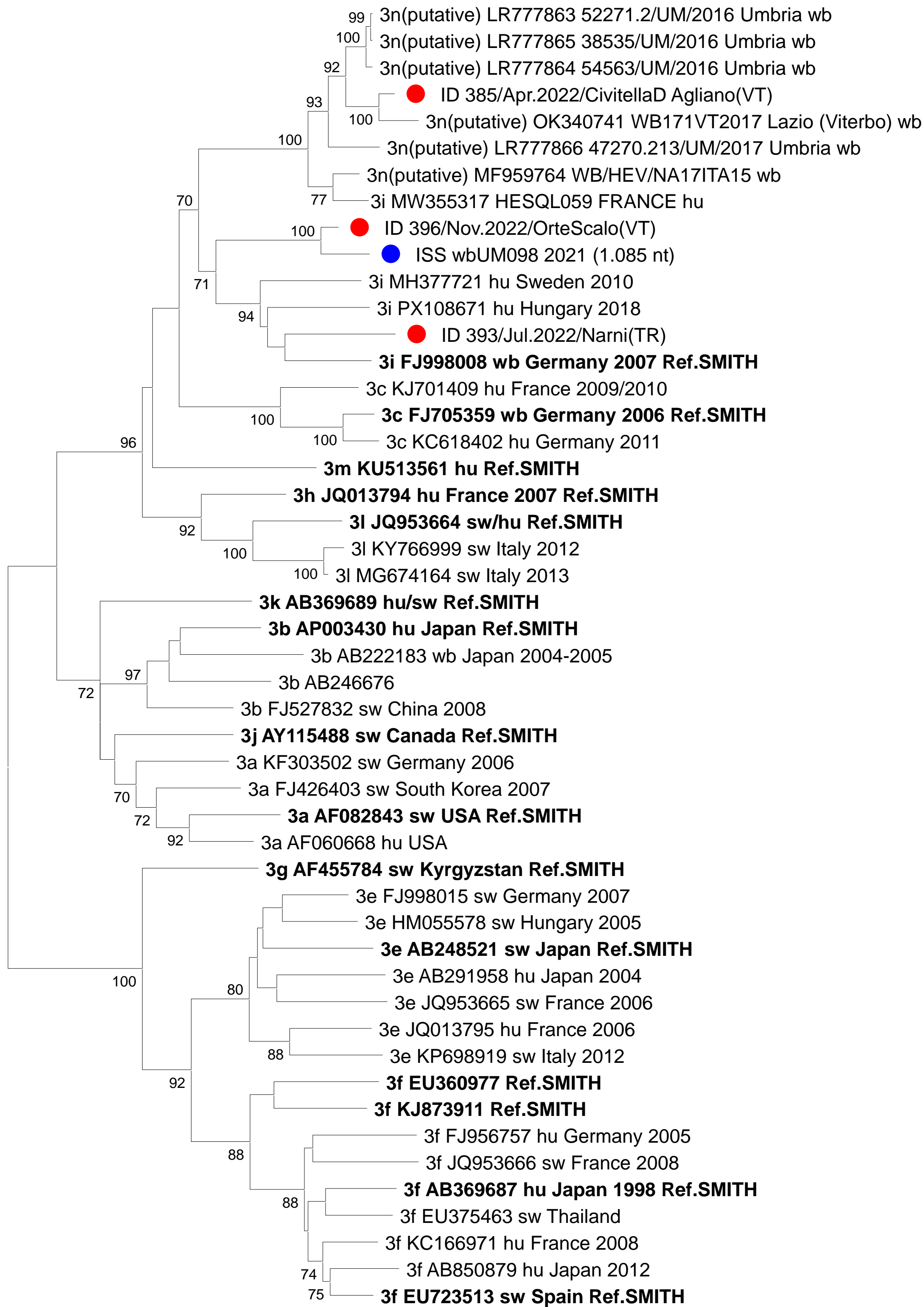

0,050

Supplement: Supplementary file 1 [file viruses-18-00709-s001.zip › viruses-4336550-supplementary.pdf]
